# Paraplegia after Apoplexy Cured by the Moxa and Nux Vomica

**Published:** 1830-01-01

**Authors:** 


### XXVII.

### PARAPLEGIA AFTER APOPLEXY REMOVED BY THE MOXA AND EXHIBITION OF NUX VOMICA.*

There can be no question but that the nux vomica is occasionally useful in paralysis, and it is equally certain that it is not unfrequently pernicious. The following case recorded by Mr. Purcell in our provincial contemporary is apparently an instance of the good effects of the drug.

*Case.* J. R. æt. 25, robust and plethoric was seized on the 8th of August, 1828, with " sanguineous apoplexy," from which under proper treatment he had recovered on the 14th. On the 16th, he complained of numbness and loss of power in the left side which were removed by cupping, but on the following day numbness and tingling were felt in the lower extremities, increased during the night, and ending next morning in complete loss of power over the limbs. Partial sensibility remained ; there was pain in the loins ; pulse 96 rather full ; bowels open by medicine ; system under the influence of mercury. He had been already cup-

* Prov. Med. Gaz. No. 11, July, 1829.

ped and blistered and the same means were now repeated with evident relief. On the 3d of September, though much improved in his general health, the loss of power in the lower extremities continued, with a partial numbness in the upper. The use of a liniment, tonic medicine, and a light nutritious diet were prescribed, but without advantage to the paraplegia, and on the 17th Mr. Purcell commenced the application of moxas to the loins every other day and the exhibition of the nux vomica. The latter remedy was at first given in the dose of a quarter of a grain of the extract, (prepared in vacuo) every six hours, and a quinine draught was taken thrice daily. On the 30th he was so much improved as to be able to stand and even walk about with the aid of crutches. The nux vomica was gradually and cautiously increased to the extent of three grains every six hours, and three or four moxas were applied daily to the loins and along the course of the principal nerves of the extremities. From the 14th of October to the 24th the patient took six grains of the extract every six hours, and at the latter date was able to walk as well as at any former period of his life. In the course of a few days he was able to resume his usual employments, the comparatively slight numbness in the upper extremities having been effectually removed by moxas along the course of the principal nerves.

This case does credit to the skill and perseverance of Mr. Purcell, and is worthy of record in these pages. The nux vomica does not seem to have produced any of its specific effects, as convulsive twitchings in the paralyzed limbs, and the dose to which the patient ultimately arrived, twenty-four grains of the extract in the day and night, was a large one. Can it be possible that the moxas had more to do with recovery than the nux vomica? This at least is certain, that the hemiplegias and paraplegias after apoplectic seizures in young men and women are not only rare occurrences, but, cæteris paribus, much more amenable to remedial measures, than palsy in persons at the turn or decline of life. This fact should be borne in mind in estimating the value of any specific drug in the former class of cases. One word in regard to the administration of the nux vomica:—we would advise practitioners to beware how they raise the dose of this formidable medicine, for it sometimes happens with it as with other poisons, that it silently accumulates in the system, and produces all at once an explosion of its effects on the constitution. We have seen convulsions and even something approaching to apoplexy ensue from its administration, and that where caution was employed.

---

